# Sparse kernel canonical correlation analysis for discovery of nonlinear interactions in high-dimensional data

**DOI:** 10.1186/s12859-017-1543-x

**Published:** 2017-02-14

**Authors:** Kosuke Yoshida, Junichiro Yoshimoto, Kenji Doya

**Affiliations:** 1 Graduate School of Informatics, Kyoto University, Kyoto, Japan; 2 Neural Computation Unit, Okinawa Institute of Science and Technology, Okinawa, Japan; 3 Graduate School of Information Science, Nara Institute of Science and Technology, Nara, Japan

**Keywords:** Kernel canonical correlation analysis, Hilbert-Schmidt independent criterion, L1 regularization

## Abstract

**Background:**

Advance in high-throughput technologies in genomics, transcriptomics, and metabolomics has created demand for bioinformatics tools to integrate high-dimensional data from different sources. Canonical correlation analysis (CCA) is a statistical tool for finding linear associations between different types of information. Previous extensions of CCA used to capture nonlinear associations, such as kernel CCA, did not allow feature selection or capturing of multiple canonical components. Here we propose a novel method, two-stage kernel CCA (TSKCCA) to select appropriate kernels in the framework of multiple kernel learning.

**Results:**

TSKCCA first selects relevant kernels based on the HSIC criterion in the multiple kernel learning framework. Weights are then derived by non-negative matrix decomposition with L1 regularization. Using artificial datasets and nutrigenomic datasets, we show that TSKCCA can extract multiple, nonlinear associations among high-dimensional data and multiplicative interactions among variables.

**Conclusions:**

TSKCCA can identify nonlinear associations among high-dimensional data more reliably than previous nonlinear CCA methods.

## Background

Canonical correlation analysis (CCA) [1] is a statistical method for finding common information from two different sources of multivariate data. This method optimizes linear projection vectors so that two random multivariate datasets are maximally correlated. With advances in high-throughput biological measurements, such as DNA sequencing, RNA microarrays, and mass spectroscopy, CCA has been extensively used for discovery of interactions between the genome, gene transcription, protein synthesis, and metabolites [2–5]. Because CCA solution is reduced to an eigenvalue problem, multiple components of interactions with sparse constraints are readily introduced [4, 6, 7].

Kernel CCA (KCCA) was introduced to capture nonlinear associations between two blocks of multivariate data [8–11]. Given two blocks of multivariate data **x** and **z**, KCCA finds nonlinear transformations *f*(**x**) and *g*(**z**) in a reproducing kernel Hilbert space (RKHS) so that the correlation between *f*(**x**) and *g*(**z**) is maximized. In order to avoid overfitting and to improve interpretability of results, sparse additive functional CCA (SAFCCA) [12] constrains *f*(**x**) and *g*(**z**) as sparse additive models and optimizes them using the biconvex back-fitting algorithm [13]. However, it is not straightforward to obtain multiple orthogonal transformations for extracting multiple components of associations. Another method for finding nonlinear associations is to maximize measures of nonlinear matching, such as the Hilbert-Schmidt Independent Criterion (HSIC) [14] and the Kernel Target Alignment (KTA) [15] between linearly projected datasets **x** and **z** [16]. While these methods can obtain multiple orthogonal projections by iteratively analyzing residuals, it is impossible for these methods to remove irrelevant features, making them prone to overfitting.

In this paper, we propose two-stage kernel CCA (TSKCCA), which enables us (1) to select sparse features in high-dimensional data and (2) to obtain multiple nonlinear associations. In the first stage, we represent target kernels with a weighted sum of pre-specified sub-kernels and optimize their weight coefficients based on HSIC with sparse regularization. In the second stage, we apply standard KCCA using target kernels obtained in the first stage to find multiple nonlinear correlations.

We briefly review CCA, KCCA, and two-stage MKL, and then present TSKCCA algorithm. We apply TSKCCA to three synthetic datasets and nutrigenomic experimental data to show that the method discovers multiple nonlinear associations within high-dimensional data, and provides interpretation that are robust to irrelevant features.

## CCA, kernel CCA, and multiple kernel learning

In this section, we briefly review the bases of our proposed method, namely, linear canonical correlation analysis (CCA), kernel CCA (KCCA), and multiple kernel learning (MKL).

### Canonical correlation analysis (CCA)

Let $D = \{ (\textbf {x}_{n}, \textbf {z}_{n}) \}_{n=1}^{N}$ be *N* pairs of samples, where **x**_*n*_ and **z**_*n*_ are the *n*-th samples drawn from *p*- and *q*-dimensional Euclidian space, respectively. Let *f*_*w*_(**x**)≡**w** ^*T*^ **x** and *g*_*v*_(**z**)≡**v** ^*T*^ **z** denote the projection of $\textbf {x} \in \mathbb {R}^{p}$ by $\textbf {w} \in \mathbb {R}^{p}$ and that of $\textbf {z} \in \mathbb {R}^{q}$ by $\textbf {v} \in \mathbb {R}^{q}$, respectively. The objective of linear CCA is to find projections that maximize Pearson’s correlation between $F_{w} \equiv \{ f_{w}(\textbf {x}_{n})\}_{n=1}^{N}$ and $G_{v} \equiv \{ g_{v}(\textbf {z}_{n})\}_{n=1}^{N}$ and formulated as the following optimization problem:

1a $$\begin{array}{@{}rcl@{}} &{\underset{{\textbf{w} \in \mathbb{R}^{p}, \textbf{v} \in \mathbb{R}^{q}}}{\max}} &~ \text{Cov}(F_{w},G_{v})  \end{array} $$

1b $$\begin{array}{@{}rcl@{}} &\text{subject to} &~ \text{Var}(F_{w}) = \text{Var}(G_{v}) = 1, \end{array} $$

where Var(·) and Cov(·,·) denote the empirical variance and covariance of the data, respectively. The optimal solution (**w** ^∗^,**v** ^∗^) of Eq. (1a and 1b) is obtained by solving generalized eigenvalue problems and successive eigenvectors represent multiple components. The projections, *f* ^∗^(**x**)=**w** ^∗*T*^ **x** and *g* ^∗^(**z**)=**v** ^∗*T*^ **z**, are said to be canonical variables for $\textbf {x} \in \mathbb {R}^{p}$ and $\textbf {z} \in \mathbb {R}^{q}$, respectively. If we introduce sparse regularization on **w** and **v**, we obtain sparse projections [4, 6, 7].

### Kernel CCA

In Kernel CCA (KCCA), we suppose that the original data are mapped into a feature space via nonlinear functions. Then linear CCA is applied in the feature space. More specifically, nonlinear functions $\phi _{x}: \mathbb {R}^{p} \to \mathbb {H}_{x}$ and $\phi _{z}: \mathbb {R}^{q} \to \mathbb {H}_{z}$ transform the original data $\{(\textbf {x}_{n}, \textbf {z}_{n})\}_{n=1}^{N}$ to feature vectors $\{(\phi _{x}(\textbf {x}_{n}), \phi _{z}(\textbf {z}_{n}))\}_{n=1}^{N}$ in reproducing kernel Hilbert spaces (RKHS) $\mathbb {H}_{x}$ and $\mathbb {H}_{z}$. Inner-product kernels for $\mathbb {H}_{x}$ and $\mathbb {H}_{z}$ are defined as *k*_*x*_(**x**,**x** ^′^)=*ϕ*_*x*_(**x**)^*T*^ *ϕ*_*x*_(**x** ^′^), and *k*_*z*_(**z**,**z** ^′^)=*ϕ*_*z*_(**z**)^*T*^ *ϕ*_*z*_(**z** ^′^).

Let us implement *f*_*w*_(**x**) and *g*_*v*_(**z**) by projections *f*_*w*_(**x**)≡**w** ^*T*^ *ϕ*_*x*_(**x**) and *g*_*v*_(**z**)≡**v** ^*T*^ *ϕ*_*z*_(**z**). By introducing appropriate regularization terms, Eq. (1a and 1b) can be reformulated as the following optimization problem ([8, 9]):

2a $$\begin{array}{@{}rcl@{}}  &{\underset{\alpha \in \mathbb{R}^{N},\beta \in \mathbb{R}^{N}}{\max}} &~ \alpha^{T} K_{x} K_{z} \beta \end{array} $$

2b $$\begin{array}{@{}rcl@{}} &\text{subject to} &~\boldsymbol{\alpha}^{T} \left(K_{x}+ \frac{N \kappa}{2} I \right)^{2}\boldsymbol{\alpha}=1  \end{array} $$

2c $$\begin{array}{@{}rcl@{}} &&~\boldsymbol{\beta}^{T} \left(K_{z} + \frac{N \kappa}{2} I \right)^{2}\boldsymbol{\beta}=1, \end{array} $$

where *K*_*x*_ and *K*_*z*_ are *N*-by-*N* kernel matrices defined as $\phantom {\dot {i}\!}[K_{x}]_{nn'} = k_{x}(\textbf {x}_{n},\textbf {x}_{n'})$ and $\phantom {\dot {i}\!}[K_{z}]_{nn'} = k_{z}(\textbf {z}_{n},\textbf {z}_{n'})$ ^1^. *I* is the *N*-by-*N* identity matrix and *κ* (*κ*>0) is the regularization parameter.

Once having obtained the solution of Eq. (2a–2c), denoted by (***α*** ^∗^,***β*** ^∗^), canonical variables for $\textbf {x} \in \mathbb {R}^{p}$ and $\textbf {z} \in \mathbb {R}^{q}$ are given by

3a $$\begin{array}{*{20}l} f^{\ast}(\textbf{x}) &= \sum_{n=1}^{N} k_{x}(\textbf{x},\textbf{x}_{n}) \alpha^{\ast}_{n} \end{array} $$

3b $$\begin{array}{*{20}l} g^{\ast}(\textbf{z}) &= \sum_{n=1}^{N} k_{z}(\textbf{z},\textbf{z}_{n})\beta^{\ast}_{n}, \end{array} $$

respectively. As indicated by Eq. (2a–2c), the nonlinear functions, *ϕ*_*x*_ and *ϕ*_*z*_, are not explicitly used in the computation of KCCA. Instead, the kernels *k*_*x*_ and *k*_*z*_ implicitly specify the nonlinear functions, and the main goal is to solve the constrained quadratic optimization problem with 2*N*-dimensional variables.

### Multiple kernel learning

Kernel methods usually require users to design a particular kernel, which critically affects the performance of the algorithm. To make the design more flexible, the framework of multiple kernel learning (MKL) was proposed for classification and regression problems [17, 18]. In MKL, we manually design *M*_*x*_ sub-kernels $\{ k_{x}^{(m)}\}_{m=1}^{M_{x}}$, where each sub-kernel $k_{x}^{(m)}$ uses only a distinct set of features in **x**. Also, *M*_*z*_ sub-kernels $\{ k_{z}^{(l)}\}_{l=1}^{M_{z}}$ for **z** is also designed in the same manner. Then, *k*_*x*_ and *k*_*z*_ are represented as the weighted sum of those sub-kernels:

4a $$\begin{array}{*{20}l} k_{x}(\textbf{x},\textbf{x}') &= \sum_{m=1}^{M_{x}} \eta_{m} k_{x}^{(m)}(\textbf{x},\textbf{x}') \end{array} $$

4b $$\begin{array}{*{20}l} k_{z}(\textbf{z},\textbf{z}') &= \sum_{l=1}^{M_{z}} \mu_{l} k_{z}^{(l)}(\textbf{z},\textbf{z}'), \end{array} $$

where weight coefficients of sub-kernels, $\{ \eta _{m} \}_{m=1}^{M_{x}}$ and $\{ \mu _{l} \}_{l=1}^{M_{z}}$ are tuned to optimize an objective function.

A specific example of this framework is the two-stage MKL approach [15, 19]: In the first stage, the weight coefficients are optimized based on a similarity criterion, such as the kernel target alignment; then, a standard kernel algorithm, such as support vector machine, is applied in the second stage.

## Methods

In this section, we propose a novel nonlinear CCA method, two-stage kernel CCA (TSKCCA), inspired by the concepts of sparse multiple kernel learning and kernel CCA. In the following, we present the general framework of TSKCCA, followed by our solutions for practical issues in the implementation.

### First stage: multiple kernel learning with HSIC and sparse regularizer

In TSKCCA, sub-kernels are restricted to the same class as Eq. (4a and 4b), allowing us to express the kernel matrices *K*_*x*_ and *K*_*z*_ as

5a $$\begin{array}{*{20}l} K_{x} &= \sum_{m=1}^{M_{x}} \eta_{m} K_{x}^{(m)} \end{array} $$

5b $$\begin{array}{*{20}l} K_{z} &= \sum_{l=1}^{M_{z}} \mu_{l} K_{z}^{(l)}, \end{array} $$

where $[K_{x}^{(m)}]_{nn'} = k_{x}^{(m)}(\mathbf {x}_{n},\mathbf {x}_{n'})$ and $[K_{z}^{(l)}]_{nn'} = k_{z}^{(l)}(\mathbf {z}_{n},\mathbf {z}_{n'})$. The goal of the first stage is to optimize the weight vector $\phantom {\dot {i}\!}\boldsymbol {\eta }=(\eta _{1},\ldots,\eta _{M_{x}})^{T}$ and $\phantom {\dot {i}\!}\boldsymbol {\mu }=(\mu _{1},\ldots,\mu _{M_{z}})^{T}$ so that kernel matrices *K*_*x*_ and *K*_*z*_ statistically depend on each other as much as possible, while irrelevant sub-kernels are filtered out.

The statistical dependence between *K*_*x*_ and *K*_*z*_ is evaluated by the Hilbert-Schmidt Independent Criterion (HSIC) and approximated by its empirical estimator [14]:

6 $$ \mathbb{D}(K_{x},K_{z}) =\frac{\text{Tr}(K_{x} H K_{z} H)}{(N-1)^{2}},  $$

where *H* is an *N*-by-*N* matrix such that $[H]_{nn'} = \delta _{nn'} - \frac {1}{N}$, and $\phantom {\dot {i}\!}\delta _{nn'}$ is Kronecker’s delta. Tr(·) denotes the trace. In our setting, optimization problem is reduced to a simple biliear form with respect to *η* and *μ*:

7 $$ \mathbb{D}(K_{x},K_{z})= \boldsymbol{\eta}^{\mathrm{T}} M \boldsymbol{\mu},  $$

where *M* is a *M*_*x*_-by- *M*_*z*_ matrix such that

8 $$ [M]_{ml}=\frac{\text{Tr}(K_{x}^{(m)} H K_{z}^{(l)}H)}{(N-1)^{2}}.   $$

In addition to maximizing the dependency measure $\mathbb {D}(K_{x},K_{z})$, ***η*** and ***μ*** should be sparse in order to filter out irrelevant sub-kernel matrices. To this end, we determine optimal weight vectors as the solution of the following constrained optimization problem:

9a $$\begin{array}{*{20}l} {\underset{\boldsymbol{\eta} \in \mathbb{R}^{M_{x}},\boldsymbol{\mu} \in \mathbb{R}^{M_{z}}}{\max}} &~ \mathbb{D}(K_{x},K_{z})=\boldsymbol{\eta}^{\mathrm{T}} M\boldsymbol{\mu} \end{array} $$

9b $$\begin{array}{*{20}l} \text{subject to} &~ \boldsymbol{\eta} \geq 0,~\boldsymbol{\mu} \geq 0, \\ &~\left\| \boldsymbol{\eta} \right\|_{2} = \left\| \boldsymbol{\mu} \right\|_{2} = 1, \end{array} $$

9c $$\begin{array}{*{20}l} &~\left\| \boldsymbol{\eta} \right\|_{1} \leq c_{1},\left\| \boldsymbol{\mu} \right\|_{1} \leq c_{2}, \end{array} $$

where $\left \| \mathbf {x}\right \|_{p} = (\sum _{i} |x_{i}|^{p})^{1/p} $ is the *L* ^*p*^-norm of the vector **x** and *c*_1_ and *c*_2_ are parameters (See also “Parameter tuning by a permutation test” section). This optimization problem is an example of penalized matrix decomposition with non-negativity constraints [4]. Accordingly, we can obtain optimal weight coefficients by performing singular value decomposition of matrix *M* under constraints. In this process, the *i*-th left singular vector $\boldsymbol {\eta }^{(i)} = (\eta _{1}^{(i)}, \dots, \eta _{M_{x}}^{(i)})^{T}$ as well as the right singular vector $\boldsymbol {\mu }^{(i)} = (\mu _{1}^{(i)}, \dots, \mu _{M_{z}}^{(i)})^{T}$ are obtained iteratively by Algorithm 1.



In Algorithm 1, *S* denotes the element-wise soft-thresholding operator: The *m*-th element of *S*(**a**,*c*) is given by sign(*a*_*m*_)(|*a*_*m*_|−*c*)_+_, where (*x*)_+_ is *x* if *x*≥0 and 0 if *x*<0. In each step, *Δ* is chosen by a binary search so that L1 constraints ∥***η***∥_1_≤*c*_1_ and ∥***μ***∥_1_≤*c*_2_ are satisfied. In general, the above iteration does not necessarily converge to a global optimum. For each iteration, we initialize ***η*** ^(*i*)^ with a non-sparse, left singular vector of *M*, following the previous study, to obtain reasonable solutions [4].

### The second stage: kernel CCA

After learning kernels via penalized matrix decomposition as above, we perform the second stage of standard kernel CCA [8, 9] to obtain optimal coefficients ***α*** ^∗^ and ***β*** ^∗^ (Eq. 3a and 3b) with parameter *κ* for each pair of singular vectors $\{ \boldsymbol {\eta }^{(i)}, \boldsymbol {\mu }^{(i)}\}_{i=1}^{rank(M)}$. Given test kernel $\{ K_{x,test}^{(m)} \}_{m=1}^{M_{x}}$ and $\{ K_{z,test}^{(l)} \}_{l=1}^{M_{z}}$, test correlation corresponding to the *i*-th singular vectors is defined as correlation between $\sum _{m=1}^{M_{x}} \eta _{m} K_{x,test}^{(m)} \boldsymbol {\alpha }^{\ast }$ and $\sum _{l=1}^{M_{z}} \mu _{l} K_{z,test}^{(l)} \boldsymbol {\beta }^{\ast }$.

### Practical solutions for TSKCCA implementation

TSKCCA still has several options for sub-kernels to be designed manually. In this study, we focus on feature-wise kernel and pair-wise kernel defined in the following sections.

#### Feature-wise kernel

Feature-wise kernel was introduced to perform feature-wise nonlinear Lasso [20]. In the previous study, using feature-wise kernels as sub-kernels in sparse MKL resulted in sparsity in terms of features since each sub-kernel corresponds to each feature. With *x*_*nm*_ and *z*_*nl*_ representing the *m*-th feature for **x**_*n*_ and *l*-th feature for **z**_*n*_, respectively, we adopt the following Gaussian kernel in this study:

10a $$\begin{array}{*{20}l} [K_{x}^{(m)}]_{nn'}&= \exp \left\{ - \gamma_{x} (x_{nm}-x_{n'm})^{2} \right\} \end{array} $$

10b $$\begin{array}{*{20}l} [K_{z}^{(l)}]_{nn'}&= \exp \left\{ - \gamma_{z} (z_{nl}-z_{n'l})^{2} \right\}, \end{array} $$

where *γ*_*x*_ and *γ*_*z*_ are width parameters. By applying feature-wise kernels, projection functions are restricted to additive models defined as $f^{\ast }(\textbf {x}) = \sum _{m=1}^{p} f_{m}(\textbf {x}_{.m})$ and $g^{\ast }(\textbf {z}) = \sum _{l=1}^{q} g_{l}(\textbf {z}_{.l})$, where $f_{m}: \mathbb {R} \to \mathbb {R}$ (*m*=1,…,*p*) and $g_{l}: \mathbb {R} \to \mathbb {R}$ (*l*=1,…,*q*) are certain nonlinear functions ^2^. Note that the number of sub-kernels, *M*_*x*_ and *M*_*z*_, are equivalent to the number of features, *p* and *q*, respectively.

#### Pair-wise kernel

We introduce pair-wise kernels as sub-kernels to consider cross-feature interactions among all possible pairs of features. Since the sparseness is induced to the weight of sub-kernels, the pair-wise kernels result in selecting relevant cross-feature interactions. Projection functions are defined as $f^{\ast }(\textbf {x}) = \sum _{m < m'}^{p} f_{m,m'}(\textbf {x}_{.m},\textbf {x}_{.m'})$ and $g^{\ast }(\textbf {z}) = \sum _{l < l'}^{q} g_{l,l'}(\textbf {z}_{.l},\textbf {z}_{.l'})$, where $f_{m,m'}: \mathbb {R}^{2} \to \mathbb {R}$ and $g_{l,l'}: \mathbb {R}^{2} \to \mathbb {R}$ are certain nonlinear functions with two dimensional inputs. Note that the number of sub-kernels, *M*_*x*_ and *M*_*z*_, are, *p*(*p*−1)/2 and *q*(*q*−1)/2, respectively.

### Preprocessing for MKL

We normalize the sub-kernels to have uniform variance in RKHS. This is an important procedure in the context of MKL because each feature-wise kernel has a different scale. This makes it difficult to evaluate weight coefficients [21]. To compensate for that, we calculate the variance *σ* ^2^ in RKHS as

11a $$\begin{array}{*{20}l} \sigma^{2}&=\frac{1}{N}\sum_{n}^{N}\left\| \phi\left(\textbf{x}_{n}\right)-\frac{1}{N}\sum_{n'}^{N} \phi\left(\textbf{x}_{n'}\right) \right\|_{2}^{2} \end{array} $$

11b $$\begin{array}{*{20}l} &=\frac{1}{N}\sum_{n}^{N}\phi\left(\textbf{x}_{n}\right)^{T}\phi\left(\textbf{x}_{n}\right)-\frac{1}{N^{2}}\sum_{n,n'}^{N}\phi \left(\textbf{x}_{n'}\right)^{T}\phi\left(\textbf{x}_{n'}\right) \end{array} $$

11c $$\begin{array}{*{20}l} &=\frac{1}{N}\sum_{n}^{N} [K]_{nn}-\frac{1}{N^{2}}\sum_{n,n'}^{N}[K]_{nn'}. \end{array} $$

Dividing each sub-kernel by its variance $K \rightarrow \frac {K}{\sigma ^{2}}$, we can achieve normalization of each sub-kernel.

### Parameter tuning by a permutation test

When the kernel matrix *K*_*x*_ (or *K*_*y*_) is full rank, as is typically our case, KCCA with a small *κ* (*κ*≪1) can always find a solution such that the maximum canonical correlation nearly equals one. This property makes it difficult to tune the regularization parameters for the first stage *c*_1_ and *c*_2_. To solve the issue, we introduce a simple heuristics.

The key idea is to conduct a permutation test for deciding whether to reject a null hypothesis that the maximal canonical correlation induced by *i*-th singular vectors is no more than those attained when **x** and **z** are statistically independent. Since the *p*-value of this test is interpreted as the deviance between the actual outcome and those expected under the null hypothesis, we use it as a score to evaluate the significance of *i*-th singular vectors where smaller *p*-value is more significant.

Algorithm 2 summarizes our implementation for the permutation test. Only for the first singular vectors ***η*** ^(1)^ and ***μ*** ^(1)^, this procedure is applied to various pairs of (*c*_1_,*c*_2_) that satisfy the constraints of $1 \leq c_{1} \leq \sqrt {M_{x}}$ and $1 \leq c_{2} \leq \sqrt {M_{y}}$ [4]. Among them, the pair with the lowest *p*-value is chosen as the optimal parameters of *c*_1_ and *c*_2_.



For simplicity, other parameters, such as *γ* in the Gaussian kernel and *κ* in KCCA, are fixed heuristically. *γ* ^−1^ is set to the median of the Euclidean distance between data points and *κ* is set to 0.02 as recommended in the previous study [9].

## Results

In this section, we experimentally evaluate the performance of our proposed TSKCCA, SAFCCA [12], and other methods using synthetic data and nutrigenomic experimental data.

### Dataset 1: single nonlinear association

To evaluate the ability to extract a single nonlinear association, we generated simple synthetic data which consisted of a single pair of relevant features in quadratic association and noise, in which standard CCA and KCCA are known to performance poorly [12]. Let *N*(*μ*,*s* ^2^) and $U({\mathcal {A}})$ denote the normal distribution with mean *μ*, variance *s* ^2^, and uniform distribution supported in ${\mathcal {A}}$, respectively. The synthetic data were generated as

$$\begin{array}{@{}rcl@{}} \textbf{x}_{.m}& \sim & U([-0.5,0.5]) \quad m=1,\ldots,D \\ \textbf{z}_{.1} &=& \textbf{x}_{.1}^{2}+\boldsymbol{\epsilon} \\ \textbf{z}_{.l}& \sim & U([-0.5,0.5]) \quad l=2,\ldots,D \\ \boldsymbol{\epsilon} & {\sim} & N(0,s^{2}), \end{array} $$

where *D* was the total number of dimensions and *ε* was independent noise.

The optimal model in each method was trained using *N* training samples. Here, we assumed *c*_1_=*c*_2_ in the range of $1 \leq c_{1}, c_{2} \leq \frac {\sqrt {D}}{2}$ and obtained optimal values using a permutation test with *B*=100. The test correlation was evaluated with separate 100 test samples, averaged over 100 simulation runs as we varied the number of dimensions, the sample size, and the noise level.

**Fig. 1:**
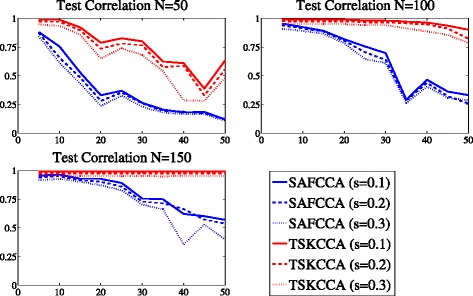
Comparison of test correlation averaged over simulation runs in Data 1. The horizontal axis denotes the number of dimensions *D*, and the vertical axis denotes test correlations. The number of training samples is 50, 100, and 150. TSKCCA outperforms SAFCCA, especially with high-dimensional data

Figure 1 shows the test correlations achieved by TSKCCA and SAFCCA with different data dimensions *D*, sample size *N*, and noise level *s*. In the first stage, our method selected only two sub-kernels, corresponding to **x**_1_ and **z**_1_, among 2×*D* sub-kernels in the first stage, especially in the case of *N*=100 and *N*=150. As a result, it achieved better test correlation than SAFCCA, especially with high-dimensional data, indicating that our method was sufficiently robust.

**Fig. 2:**
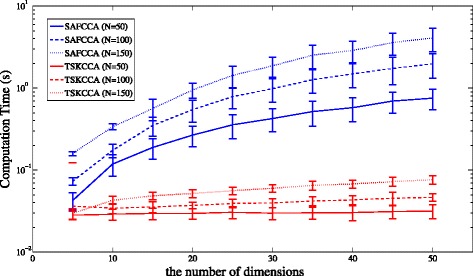
Comparison of computation time for Data 1. The horizontal axis denotes the number of dimensions *D*, and the vertical axis denotes computation time in log-scale. The number of training samples is 50, 100, 150 for SAFCCA and TSKCCA. Computation time of TSKCCA is moderate and can be scaled

In addition, Fig. 2 shows average computation time for each method over 100 simulation runs with dataset 1. Computation time of TSKCCA was comparable with that of SAFCCA, and could scale up with the feature size. Note that all the experiments were performed on a MacBook Pro with Intel Core i7 (2.9 GHz dual core processor with 4 MB L3 cache) with 8 GB main memory. All the simulation programs were implemented in MATLAB ^®;^.

### Dataset 2: multiple nonlinear associations

To test whether our method could extract multiple nonlinear associations precisely, we generated the following data:

$$\begin{array}{@{}rcl@{}} \textbf{x}_{.m}& \sim & U([-0.5,0.5]) \quad m=1,\ldots,25 \\ \textbf{z}_{.1} &=& \textbf{x}_{.1}+\exp(-\textbf{x}^{2}_{.4})+\boldsymbol{\epsilon}_{1} \\ \textbf{z}_{.2} &=& \textbf{x}_{.2}^{2}+\sin(\pi \textbf{x}_{.5}/2) +\boldsymbol{\epsilon}_{2} \\ \textbf{z}_{.3} &=& |\textbf{x}_{.3}|+1/(1+\exp(-5\textbf{x}_{.6}))+\boldsymbol{\epsilon}_{3} \\ \textbf{z}_{.l}& \sim & U([-0.5,0.5]) \quad l=4,\ldots,25 \\ \boldsymbol{\epsilon}_{l} & {\sim} & N(0,0.1^{2}) \quad l=1,2,3. \end{array} $$

First, we performed a permutation test with *B*=1000 for ten singular vectors $\{ \boldsymbol {\eta }^{(i)},\boldsymbol {\mu }^{(i)}\}_{i=1}^{10}$ corresponding to the ten highest singular values of *M* given by Eq. (8). *P*-values of the top three were significant (*p*<0.001) and the rest were non-significant. This result suggests that only the three singular vectors included nonlinear associations.

**Fig. 3:**
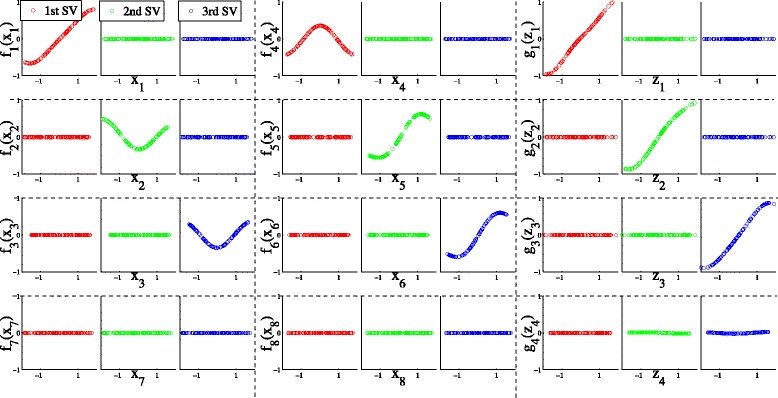
Transformations *f*(**x**) and *g*(**z**) obtained with TSKCCA. The top three rows and the bottom row show the resulting functions corresponding to relevant and irrelevant features, respectively

Figure 3 shows the transformations *f*(**x**) and *g*(**z**) obtained with TSKCCA. In the first singular vectors, the contributions of $\eta ^{1}_{1}$, $\eta ^{1}_{4}$ and $\mu ^{1}_{1}$ were dominant, indicating that **x**_.1_,**x**_.4_ and **z**_.1_ were associated. The contributions of $\eta ^{2}_{2}$, $\eta ^{2}_{5}$ and $\mu ^{2}_{2}$ in the second singular vectors were also dominant, indicating that **x**_.2_, **x**_.5_ and **z**_.2_ were associated. Finally, the contributions of $\eta ^{3}_{3}$, $\eta ^{3}_{6}$ and $\mu ^{3}_{3}$ in the third singular vectors were dominant, indicating that **x**_.3_, **x**_.6_ and **z**_.3_ were associated. Some singular vectors averaged over 100 simulation runs are listed in Table 1. Our results suggest that TSKCCA achieved feature selection precisely.

**Table 1:** Feature selection through singular vectors (SVs) in data 2

	1st SV (*η* ^(1)^)	2nd SV (*η* ^(2)^)	3rd SV (*η* ^(3)^)
*η*_1_	**0.98 (0.002)**	0.00 (0.018)	0.00 (0.001)
*η*_2_	0.00 (0.003)	**0.21 (0.033)**	0.00 (0.001)
*η*_3_	0.00 (0.001)	0.00 (0.010)	**0.22 (0.029)**
*η*_4_	**0.22 (0.013)**	0.00 (0.017)	0.00 (0.005)
*η*_5_	0.00 (0.000)	**0.98 (0.004)**	0.00 (0.005)
*η*_6_	0.00 (0.004)	0.00 (0.002)	**0.98 (0.003)**
	1st SV (*μ* ^(1)^)	2nd SV (*μ* ^(2)^)	3rd SV (*μ* ^(3)^)
*μ*_1_	**0.99 (0.005)**	0.01 (0.022)	0.01 (0.014)
*μ*_2_	0.01 (0.027)	**0.99 (0.004)**	0.01 (0.015)
*μ*_3_	0.01 (0.024)	0.01 (0.018)	**0.99 (0.003)**
*μ*_4_	0.01 (0.023)	0.01 (0.026)	0.01 (0.017)

These results show mean weight coefficients (standard deviation) in 100 simulation runs. Significant weight coefficients are bold faced

We further evaluated test correlation, precision, and recall averaged over 20 simulation runs. Table 2 shows that SAFCCA failed to detect all relevant features because it is not able to obtain multiple canonical correlations, while our method detected 9 relevant sub-kernels out of 50 in the first stage in most runs. Note that the precision is the fraction of retrieved features that are relevant and recall is the fraction of relevant features that are retrieved.

**Table 2:** Comparison of test correlation, precision, and recall in data 2

	Correlation	Precision	Recall
TSKCCA	0.9670	0.9163	1
	0.9636		
	0.9732		
SAFCCA	0.7585	0.6350	0.4375

TSKCCA can identify most relevant features through three significant singular vectors, while SAFCCA can only identify a small set of them

### Dataset 3: feature interactions

To assess the capability of TSKCCA in discovering nonlinear interactions, we generated data with a product term:

$$\begin{array}{@{}rcl@{}} \textbf{x}_{.m}& \sim & U([-0.5,0.5]) \quad m=1,\ldots,D \\ \textbf{z}_{.1} &=& \textbf{x}_{.1}\textbf{x}_{.2}+\boldsymbol{\epsilon}\\ \textbf{z}_{.l}& \sim & U([-0.5,0.5]) \quad l=2,\ldots,D \\ \boldsymbol{\epsilon} & {\sim} & N(0,0.1^{2}), \end{array} $$

where *D* was the number of dimensions. For this dataset, we used feature-wise kernels and pair-wise kernels as sub-kernels in order to handle both single feature effects and cross-feature interactions like the term **x**_.1_ **x**_.2_. There were *D*+*D*×(*D*−1)/2 sub-kernels, the weight coefficients of which were optimized in our method.

**Fig. 4:**
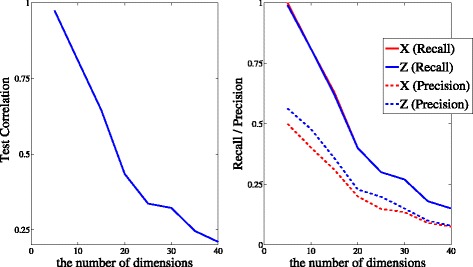
The performance of pair-wise kernels in Data 3. (*Left*) Test correlations averaged over 100 simulation runs in different numbers of dimensions. (*Right*) Recall and precision averaged over 100 simulation runs in different numbers of dimensions. Our method successfully extracts nonlinear associations with relevant features

First, to evaluate the performance of our method with feature-wise and pair-wise kernels, we obtained test correlations evaluated by individual test data (*N*=100) in different numbers of dimensions *D*. Next, to evaluate the accuracy of feature selection of the model, we assessed recall and precision. Average test correlations, recall, and precision over 100 simulation runs are shown in Fig. 4. Our results illustrate that in the case of *D*<10 (i.e. the number of sub-kernels is less than 10+10×9/2=55), our method successfully determined the relation between **z**_.1_ and **x**_.1_ **x**_.2_.

### Dataset 4: nutrigenomic data

We then analyzed a nutrigenomic dataset from a previous mouse study [22, 23]. In this study, expression of 120 genes in liver cells that would be relevant in the context of nutrition and concentrations of 21 hepatic fatty acids were measured on 20 wild-type mice and 20 PPAR *α*-deficient mice. Mice of each genotype were fed 5 different diets with different levels of fat. For matrix notation, gene expression data were denoted by $X \in \mathbb {R}^{40\times 120}$, and data regarding concentrations of fatty acids was denoted by $Z \in \mathbb {R}^{40\times 21}$. Data were standardized to have a mean of zero and unit variance in each dimension. Several linear correlations between *X* and *Z* were detected by applying a regularized version of the linear CCA [5, 23].

First, we performed a permutation test for sparse CCA, KCCA, SAFCCA, and TSKCCA on parameters defined by equally-spaced grid points in order to identify significant associations in these data. In KCCA and SAFCCA, there were no significant associations; thus, we focused on sparse CCA and TSKCCA in the following analysis. We identified two significant linear associations in sparse CCA (*p*<0.001 using a permutation test) and one nonlinear association in TSKCCA (*p*=0.0067 using a permutation test) with *c*_1_=2.6257 and *c*_2_=1.9275.

**Fig. 5:**
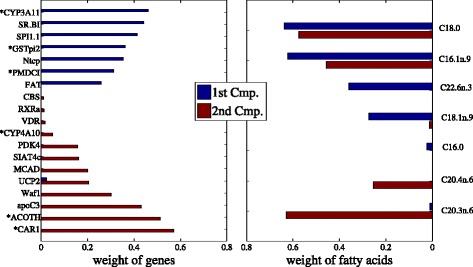
Feature selection of sparse CCA in nutrigenomic data. *Left* and *right panels* show selected genes and fatty acids, respectively. Genes marked with asterisks show significantly different expression in different genotypes

**Fig. 6:**
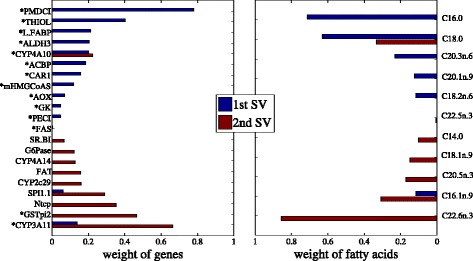
Feature selection of TSKCCA using nutrigenomic data. *Left* and *right panels* show selected genes and fatty acids, respectively. Genes marked with asterisks show significantly different expression in different genotypes. The left panel shows that the 1st singular vector extracts nonlinear correlations associated with the genotype

Figures 5 and 6 show the results of feature selection of sparse CCA and TSKCCA, respectively. Genes selected by the first singular vector of our method have different expression levels in different genotypes (marked with asterisk), suggesting that our method successfully extracted the nonlinear correlation associated with genotypes.

**Fig. 7:**
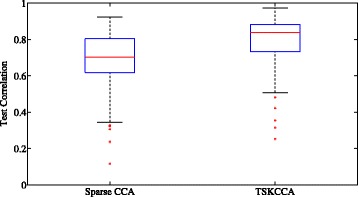
Box plot of test correlations in nutrigenomic data. *Left* and *right panels* show the box plot of 100 times test correlation using sparse CCA and TSKCCA, respectively. TSKCCA achieves significantly higher test correlation through its first weight vector (*p*<10^−6^ with a t-test)

For further analysis, cross-validation was performed in 100 runs. In each run, 40 samples were randomly split into 30 training samples used for fitting models and 10 validation samples used for evaluating the canonical correlation for fitted models. Figure 7 shows box plots of correlation coefficients in sparse CCA and TSKCCA. Left one represents the first canonical correlation coefficient in sparse CCA and right one represents correlation coefficient obtained with the first singular vectors. Significantly higher test correlation (*p*<10^−6^ with a t-test) were achieved by the first singular vectors of TSKCCA, indicating that it avoided overfitting despite having nonlinearity.

To account for interactions between features into our model, we calculated pair-wise kernels for nutrigenomic data. Although the number of sub-kernels was huge (120+120×119/2=7260 sub-kernels for genes, 21+21×20/2=231 sub-kernels for fatty acids), TSKCCA successfully extracted a significant association (*p*<0.001 using a permutation test). To evaluate the stability of feature selection, we performed TSKCCA on 1000 runs with data generated by random sampling of empirical data with replacement. Table 3 shows the frequencies of features (i.e. pairs of features) selected across 1000 runs, suggesting that *PMDCI* played an important role within the interactions.

**Table 3:** Frequency of selection per sub-kernel corresponding to genes (*left*) and fatty acids (*right*) in nutrigenomic data

Genes/Pair of genes	Freq.	Fatty acids/Pair of fatty acids	Freq
PMDCI	643	C16.0-C18.0	622
CAR1-PMDCI	564	C18.0	485
PMDCI-THIOL	563	C16.0-C20.3n.6	429
ACBP-PMDCI	473	C16.0	340
L.FABP-PMDCI	451	C18.0-C20.3n.6	315
CYP4A10-PMDCI	379	-	-
CYP3A11-PMDCI	370	-	-
ALDH3-PMDCI	369	-	-
Ntcp-PMDCI	354	-	-
PMDCI-SPI1.1	347	-	-
ACOTH-PMDCI	330	-	-
PMDCI-SR.BI	306	-	-

## Discussion

Other researchers have employed the sparse additive model [13] to extend KCCA to high-dimensional problems, and have defined two equivalent formulations, such as sparse additive functional CCA (SAFCCA) and sparse additive kernel CCA (SAKCCA) [12]. The former was defined in a second order Sobolev space and solved using the biconvex back-fitting procedure. The latter, defined in RKHS, was derived by applying representer theorem to the former. Given some function $f_{m} \in \mathbb {H}_{m}$, these algorithms optimize the additive model, $f_{1} \in \mathbb {H}_{1},f_{2} \in \mathbb {H}_{2},\ldots,f_{p} \in \mathbb {H}_{p}$. In contrast, our formulation supposes an additive kernel, such as $\sum \eta _{m} K_{m}$ associated with RKHS $\mathbb {H}_{add}$ and finds correlations in this space. This approach enables us to reveal multiple components of associations.

Some problems specific to KCCA, such as choosing two parameters (i.e. regularization parameter *κ* and the width parameter *γ*) and the number of components, remain unsolved. While cross validation is applicable to set these values [24], they are fixed for simplicity in our study, based on the previous study [9].

Next, we discuss the validity of feature selection in nutrigenomic data performed using sparse CCA and TSKCCA. In the original study, the authors focused on the role of PPAR *α* as a major transcriptional regulator of lipid metabolism and determined that PPAR *α* regulates the expression of many genes in mouse liver under lower dietary fat conditions [22]. They provided a list of genes that have significantly different expression levels between wild-type and PPAR *α*-deficient mice. While only a few genes selected by sparse CCA were included in the list, 13 out of 14 genes selected with the 1st singular vector in TSKCCA were included in the list. This result shows that TSKCCA successfully extracts meaningful nonlinear associations induced by PPAR *α*-deficiency.

Moreover, in our analysis of pair-wise kernels, most of the frequently selected pairs of genes retained *PMDCI* known as a sort of enoyl-CoA isomerases involved in *β*-oxidation of polyunsaturated fatty acids. This implies that the interactions of *PMDCI* and other genes contribute to lipid metabolism in PPAR *α*-deficient mice.

Many variants of sub-kernels, such as string kernels or graph kernels, can be employed in the same framework. In the field of bioinformatics, Yamanishi et al. adopted integrated KCCA (IKCCA), which exploited the simple sum of multiple kernels to combine many sorts of biological data [11]. This technique can be improved by optimizing weight coefficients of each kernel in the frame of TSKCCA. Finally, if kernels are defined on groups of features, it enables us to perform group-wise feature selection, just like group sparse CCA [25–27]. It is beneficial to consider group-wise feature selection for biomarker detection problems.

## Conclusions

This paper proposes a novel extension of kernel CCA that we call two-stage kernel CCA, which is able to identify multiple canonical variables from sparse features. This method optimizes the sparse weight coefficients of pre-specified sub-kernels as a sparse matrix decomposition before performing standard kernel CCA. This procedure enables us to achieve interpretability by removing irrelevant features in the context of nonlinear correlational analysis.

Through three numerical experiments, we have demonstrated that TSKCCA is more useful for higher dimensional data and for extracting multiple nonlinear associations than an existing method, SAFCCA. Using nutrigenomic data, our results show that TSKCCA can retrieve information about genotype and may reveal an interactive mechanism of lipid metabolism in PPAR *α*-deficient mice.

## Endnotes

^1^ In this article, $\phantom {\dot {i}\!}[\cdot ]_{nn'}$ denotes the (*n*,*n* ^′^)-th elements of the matrix enclosed by the brackets.

^2^ In this article, **x**_.*m*_ denotes the *m*-th feature of **x**.

## Abbreviations

CCA: Canonical correlation analysis
HSIC: Hilbert-Schmidt independent criterion
KCCA: Kernel canonical correlation analysis
MKL: Multiple kernel learning
RKHS: Reproducing kernel Hilbert space
SAFCCA: Sparse additive functional canonical correlation analysis
TSKCCA: Two-stage kernel canonical correlation analysis
